# Fecal Fat Analyses in Chronic Pancreatitis Importance of Fat Ingestion before Stool Collection

**DOI:** 10.1371/journal.pone.0169993

**Published:** 2017-01-17

**Authors:** Friedemann Erchinger, Trond Engjom, Palwasha Jurmy, Erling Tjora, Odd Helge Gilja, Georg Dimcevski

**Affiliations:** 1 Department of Medicine, Voss Hospital, Haukeland University Hospital, Voss, Norway; 2 Institute of Clinical Medicine, University of Bergen, Bergen, Norway; 3 Department of Medicine, Haukeland University Hospital, Bergen, Norway; 4 Hospital Pharmacy Enterprises, South Eastern Norway Regional Health Authority, Oslo, Norway; 5 Pediatric Department, Haukeland University Hospital, Bergen, Norway; 6 Department for Clinical Science, University of Bergen, Bergen, Norway; 7 National Centre for Ultrasound in Gastroenterology, Haukeland University Hospital, Bergen, Norway; Centro Nacional de Investigaciones Oncologicas, SPAIN

## Abstract

**Objective:**

Quantitative determination of fecal fat still is the gold standard for measuring malabsorption. We evaluated the importance of standardized food intake before and under the collection of feces.

**Material and Methods:**

In a project, evaluating patients with suspected chronic pancreatitis (CP) and healthy volunteers (HC), stools were collected for 72 hours coupled to registration of nutritional intake over five consecutive days. Patient groups were created by a modified Layer score, which includes imaging findings, clinical parameters and pancreas function testing.

**Results:**

We found 12 patients with CP, 11 patients without CP and 13 healthy individuals in our database. Median fecal fat in CP patients was 12 g/day, in non-CP patients 5 g/day and in healthy controls 5 g/day. Median fat absorption coefficient was 81% in those with chronic pancreatitis, 92% in those without CP and 92% in healthy controls. Corresponding median fat intake was 65 g/day, 68 g/day and 81 g/day in the respective groups. Spearman Rank Order Correlation between fecal fat (g/d) and fat absorption coefficient in all study subjects (n = 36) was good (-0.88 (p<0.001)). When we stratified groups according to fat intake, correlation between fecal fat and fat absorption was also good (-0.86 to -0.95).

**Conclusion:**

In the diagnoses of fat malabsorption, calculating the ratio of fat absorption did not give additional information compared to fecal fat.

## Introduction

Fat malabsorption is a serious condition leading to malnutrition and deficiency of fat-soluble vitamins [1–3]. It is caused by insufficient secretions of biliary juice, pancreatic enzymes or impaired intestinal brush border. Even if not in use as a method in clinical routine, quantification of fecal fat may be a useful tool in the assessment of chronic diarrhoea to identify fat malabsorption in a broad specter of diseases. Fecal fat determination remains the gold standard in quantification of fat malabsorption even though it is performed sporadically [4–6]. Compared to the original method by van de Kamer, Berstad et al. performed measurement with 10% of feces and reagents to minimize the use of highly toxic reagents. The principals of this method can be described as follows: After hydrolysis of triglycerides, fatty acids are extracted with petroleum ether and finally quantified by titration. Evaluation of this modification showed excellent accuracy[7]. Quantification of fecal fat is inconvenient for the patient and laboratory staff, and infrequently performed. In many cases, patient adherence to a standardized five days’ diet containing 100 g/d fat is poor. Stool collection according to protocol requires highly motivated patients [5,8]. In some specialized centers, admission of patients over five days is part of the procedure to guarantee that performance is correct. However, requirements of cost effectiveness in modern hospitals limit the possibility for such an approach. A simplification of the method is highly warranted. The crucial part of fecal fat determination is the three days’ collection of stools. We hypothesize that patients can perform this at home on unadjusted fat intake over five days.

The coefficient of fat absorption (CFA) was used over the last century in many studies. However, to our knowledge there is no study which explores whether CFA is mandatory adding substantial information to the simpler measurement of fecal fat [9–11].

In this study, we aimed to explore if calculation of the fecal fat absorption coefficient with documented intake of food over five days gave clinical more relevant information than the measurement of daily fecal fat in the diagnosis of fat malabsorption.

## Materials and Methods

### Patient recruitment

**Fig 1:**
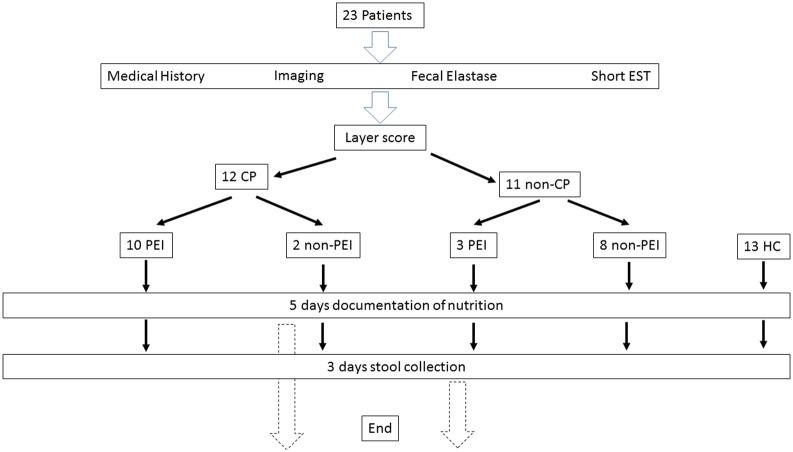
Flowchart of the retrospective study from a database for chronic pancreatitis. Short EST = Short endoscopic secretin test. The Layer score bases on imaging, pancreas function testing and clinical history. Similar to the Mayo score it differentiates between CP (chronic pancreatitis) and non-CP (= result of scoring not suggestive for chronic pancreatitis). PEI = pancreatic exocrine insufficiency. We defined gold standard for PEI as Bicarbonate concentration < 80 mmol/l in the short endoscopic secretin test as it is a direct pancreas function test. Consecutively a test result of bicarbonate concentration in duodenal juice > 80 mmol/l is not suggestive for pancreatic exocrine insufficiency (non-PEI). HC = healthy controls.

We used data from our chronic pancreatitis project. In this project, patients referred to our outpatient clinic with symptoms suspicious for chronic pancreatitis (CP) were offered participation in the study. In the original project, we recruited a group of healthy controls by advertising through board notes in two participating regional hospitals. The fecal fat collections were performed in the period from September 2010 to June 2011. Fig 1 shows a flowchart of the retrospective study protocol.

### Ethics approval and consent to participate

The protocol was approved by the Regional committee for medical and health research ethics, Western Norway (post@helseforskning.etikkom.no; approval number: REK: 3.2008.2516) and the study was performed in accordance with the Helsinki Declaration. All subjects signed an informed consent. This study is part of a project registered in clinicaltrials.gov (Identifier: NCT01059669).

### The diagnostic standard for chronic pancreatitis

Patients went through a multimodal examination algorithm including clinical information, imaging and pancreas function testing [12]. We tested pancreatic exocrine function by fecal elastase 1 and bicarbonate concentration in duodenal juice aspirated during a short endoscopic secretin test. At least one modality of imaging (CT, EUS and MRI), was either performed or obtained from the patient journal. We evaluated the patients for the diagnosis of chronic pancreatitis by a modified Layer score as described by Erchinger et al.[13]: Calcifications or histology = 4 points; imaging with either ERCP, secretin stimulated MRCP; MRCP, endosonography or transabdominal ultrasound, when results qualified for CP = 3 points (Cambridge, M-ANNHEIM or Rosemont score)[14–17]; test for pancreatic exocrine insufficiency with fecal Elastase, fecal fat or direct pancreatic function testing = 2 points; history of acute pancreatitis or classical abdominal pain over at least 6 months = 2 points; diabetes mellitus = 1 point. Scoring results created two patient groups: CP confirmed ≥ 4 points/not confirmed < 4 points (CP/non-CP). None of the patients in the non-CP group suffered from IBD or short bowel syndrome. Final diagnoses were: IBS with or without diarrhea or patients with abdominal pain of other reasons. Duodenal bicarbonate from direct pancreas function testing was reference standard in the diagnosis of PEI in the Layer score. We defined values lower than 80 mmol/l pathological. In the patient group, this definition yielded 13 patients with exocrine insufficiency. The diagnostic score was not performed on the healthy control group.

### Nutritional intake in the sampling period

All study subjects were instructed by a master grade candidate in nutrition to document intake of food and beverages over five days. All aliments were weighted and—if meals where prepared from raw materials—recipes with ingredients in detail were attached. The participants did not have to change their eating habits. The subjects stopped all enzyme supplements during the study period.

We analyzed the nutritional content of the food intake for each participant using validated tables of nutritional content in foods published by the Norwegian health authorities. The amounts of calories, fat, protein, carbohydrate and calcium, zinc and vitamin D were calculated [18]. We compared the average intake of nutrition in the study groups to the nutritional recommendations from the health authorities.

### Stool sampling and analyses

The subjects collected stools for the last three consecutive days of the five days’ period. We have previously described the procedure of stool collection, homogenization and titration. Our modified version of the titration method uses less than one tenth of the amount of faeces originally described [19].

Fecal fat was expressed in gram /day (g/d). Values >7g/d were defined as malabsorption [20,21]. Fat absorption coefficient was calculated as follows: ((Fat ingestion–Fat excretion) / Fat ingestion) x 100(%). Fat absorption coefficients <90% were defined as insufficient [3,10].

All participants also collected a specimen of stool for analyses of fecal Elastase (Pancreatic Elastase 1, Schebo Biotech AG, Giessen, Germany). A cut-off of 200 μg/g faeces was used [22].

### Statistical analyses

Median and interquartile range (IQR) expressed the results if not otherwise stated. We compared groups by t test; if normality failed, we ran a Mann Whitney Rank sum test. Power analysis was performed in advance by (https://www.dssresearch.com/KnowledgeCenter/toolkitcalculators/samplesizecalculators, DSS Research, Arlington, Washington DC, USA)) Spearman-rank calculations evaluated correlations between values. Accuracy calculations were performed by ROC analyses.

## Results

### Demographic data and parameters for pancreatic exocrine insufficiency

Table 1 displays demographic data and parameters for pancreatic exocrine insufficiency (PEI). CP patients were older than HC (p<0.001) and non-CP patients (p = 0.03). Non-CP patients were older than HC (p = 0.02). CP patients had significant lower Duodenal-Bicarbonate than non-CP patients (p<0.001) and HC (p<0.001). Fecal elastase was lower in the CP group than in HC (p<0.001) and non-CP group (<0.04). Fecal fat distinguished between the CP group and non-CP group resp. HC (p<0.047; p = 0.039). Coefficient of fat absorption showed the following: CP/HC: p = 0.007; CP/non-CP: p = 0.029. There was not any difference for diagnostic parameters between the non-CP and HC group.

**Table 1:** Demographic data and results from pancreatic function testing.

	All	Healthy Controls	CP	Non-CP
**n**	36	13	12	11
**Age** median (range)	50 (25/80)	39 (27/54)	65 (35/80)	48 (30/68)
**Female/Male** (n)	14/22	5/8	4/8	5/6
**BMI**	24 (21/26)	25 (23/31)	23 (20/26)	24 (20/26)
**Bicarbonate** (mmol/L)	95 (70/111)	114 (97/120)	51 (14/72)	100 (76/111)
**Fecal elastase** 1 (μg/g)	440 (147/596)	561 (496/629)	97 (15/426)	370 (253/650)
**Fecal fat** (g/d)	6 (4/10)	5 (4/9)	13 (5/35)	5 (2/8)
**Fat absorption** (%)	91 (84/95)	92 (91/96)	81 (36/91)	92 (86/97)

All study subjects underwent a multimodal diagnostic algorithm including clinical information, imaging and pancreas function testing. Based on this information we used a modified scoring system after Layer leading to two patient groups: CP = patients with chronic pancreatitis, non-CP = patients with abdominal symptoms suspicious for chronic pancreatitis but negative scoring for CP. CP patients were older than non-CP patients (ns) and HC. Non CP patients were older than HC. BMI: no significant difference; Gender: no significant difference. BMI = body mass index. n = numbers. Bicarbonate = bicarbonate in duodenal juice after secretin stimulation. Values in median and interquartile range unless otherwise stated.

### Nutrients

Table 2 presents ingestion of nutrients compared to recommendations of the Norwegian health authorities. In all groups, median intake of energy, fat and proteins was within the recommendations, but the median intake of carbohydrates fiber, vitamin D and Calcium of all groups were lower than advocated by the local authorities. In the non-CP and HC group, median intake of zinc was higher than recommended. There was no statistical significant difference in quantity of nutrients recorded between the subject categories.

**Table 2:** Nutrition intake.

*Ingestion*	*Recommended*	*CP*	*Non-CP*	*HC*
**Energy (Kcal/d)**	1800–2200	1835 (1396–2583)	1832 (1462–2358)	1876 (1479–2363)
**Fat (g/d)**	50–86	65 (49–84)	68 (49–89)	80 (54–102)
**Protein (g/d)**	50–110	72 (59–79)	76 (65–146)	86 (78–106)
**Carbohydrates (g/d)**	225–330	161 (152–354)	195 (137–270)	187 (137–254)
**Fiber (g/d)**	25–35	20 (13–26)	22 (16–40)	21 (16–27)
**Zinc (mg/d)**	7–9	8 (8–9)	11 (8–17)	12 (8–17)
**Calcium (mg/d)**	800	586 (391–696)	797 (470–1091)	725 (501–1242)
**D-vitamin (μg/d)**	7,5	5 (2–10)	3 (2–7)	5 (2–10)

Median and Interquartile Range of nutrients intake per day compared to recommended range of ingestion; Recommendations from Norwegian health authorities. CP = chronic pancreatitis group; non CP = non chronic pancreatitis patient group; HC = healthy controls

### Correlation of fecal fat and fat absorption coefficient

**Fig 2:**
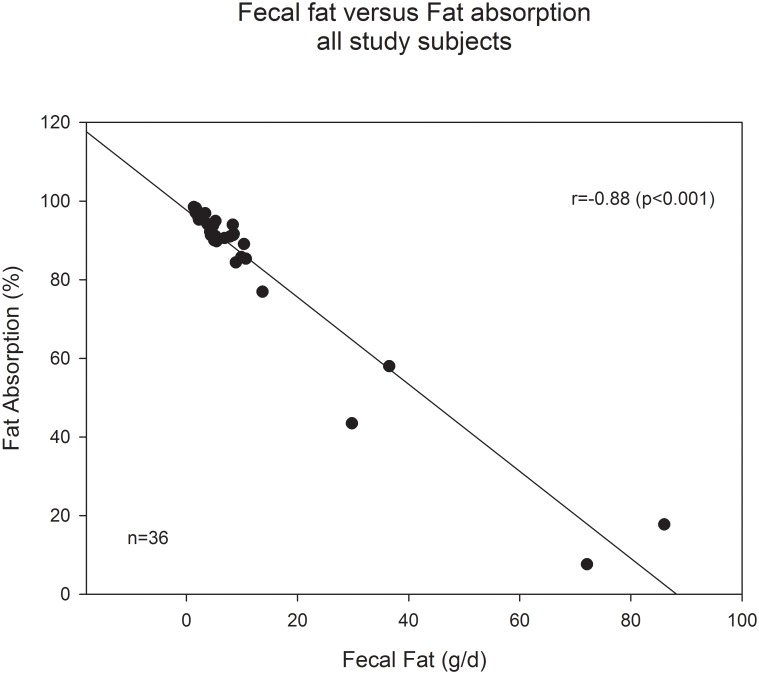
Correlation of fecal fat (g/d) vs fat absorption (%) in relation to fat intake (g/d). All study subjects.

The correlation coefficient between fecal fat and fat absorption coefficient in all study subjects (n = 36) was -0.876 (p<0.001). Absolute fat values in stool correlate well to the more complex, intake adjusted fat absorption coefficient (Fig 2).

**Fig 3:**
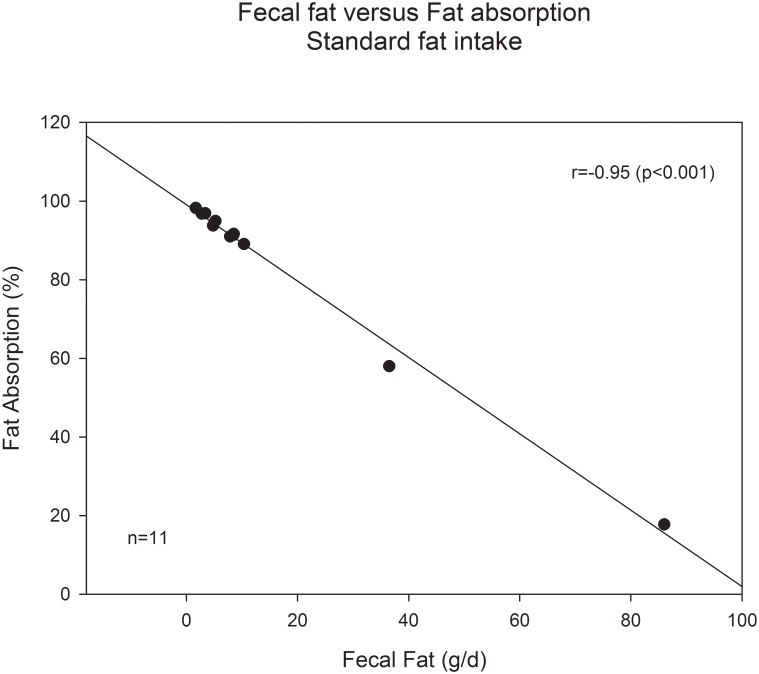
Correlation of fecal fat (g/d) vs fat absorption (%) in relation to fat intake (g/d). Recommended range of fat intake (80–120 g/d). Absolute fat values in stool correlate well to the more complex, intake adjusted fat absorption coefficient.

We also analyzed correlations between fecal fat and fat absorption in subgroups defined by nutritional fat intake. In the reference group (Fig 3) with fat intake 80–120 g, correlation coefficient was -0.945 (p<0.001, n = 11). Without the four subjects with very low (<40g/d) or high (>160g/d) fat intake the correlation coefficient was nearly as good as in the reference group: r = -0.921 (p<0.001, n = 31).

### Fecal fat parameters related to EPI standard

**Fig 4:**
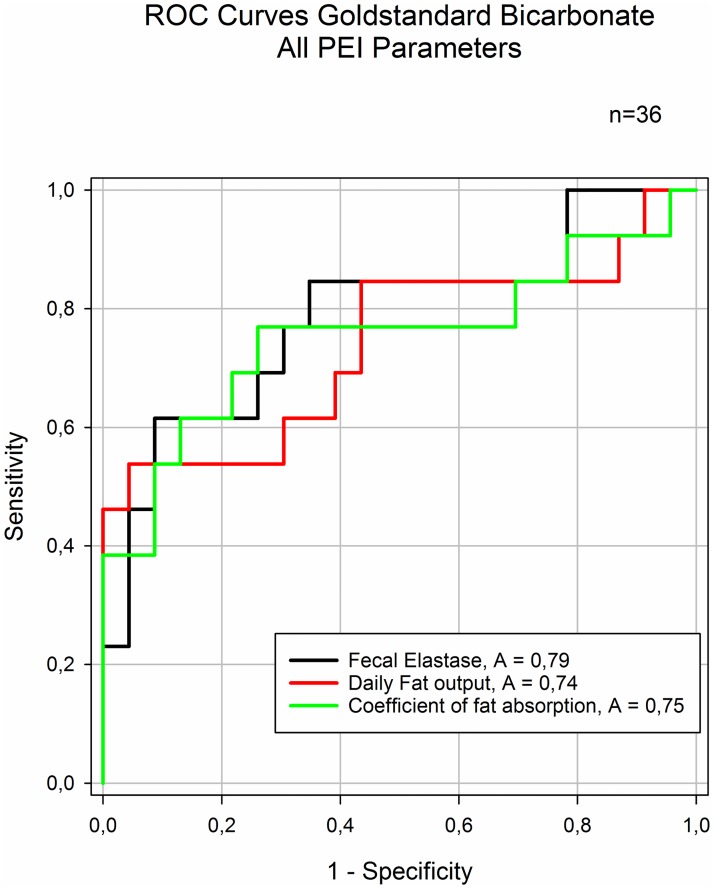
ROC curves for EPI parameters in all study subjects. As result of direct pancreas testing bicarbonate in duodenal juice after secretin stimulation was chosen as standard. No significant difference in Area under the curve (AUC) between f-Elastase and fecal fat (CI -0,15 to 0,25); f-Elastase and CFA (CI -0,19 to 0,25); fecal fat and CFA (-0,11 to 0,08) ROC curve = receiver operating characteristic curve; AUC = Area under the curve; CI = confidence interval.

Using cut-off for bicarbonate concentration of 80mmol/l as standard, we drew ROC curves for fecal elastase and the two fecal fat parameters (Fig 4). Data for diagnostic accuracy are presented for the respective parameters in Table 3. The ROC curves displayed no significant difference in diagnostic performance between the chosen parameters for the PEI standard.

**Table 3:** Accuracy for indirect exocrine pancreas function parameters against exocrine pancreatic function determined by endoscopic secretin test.

	*Sensitivity*	*CI*	*Specificity*	*CI*	*PPV*	*NPV*
**Fecal Elastase**	0,46	0,19 to 0,75	0,91	0,72 to 0,99	0,7	0,8
**Fecal Fat**	0,62	0,32 to 0,86	0,65	0,43 to 0,84	0,5	0,8
**CFA**	0,62	0,32 to 0,86	0,78	0,56 to 0,93	0,6	0,8

CI = confidence interval, PPV = positive predictive value, NPV = negative predictive value.

### Fecal fat parameters related to CP diagnosis

**Fig 5:**
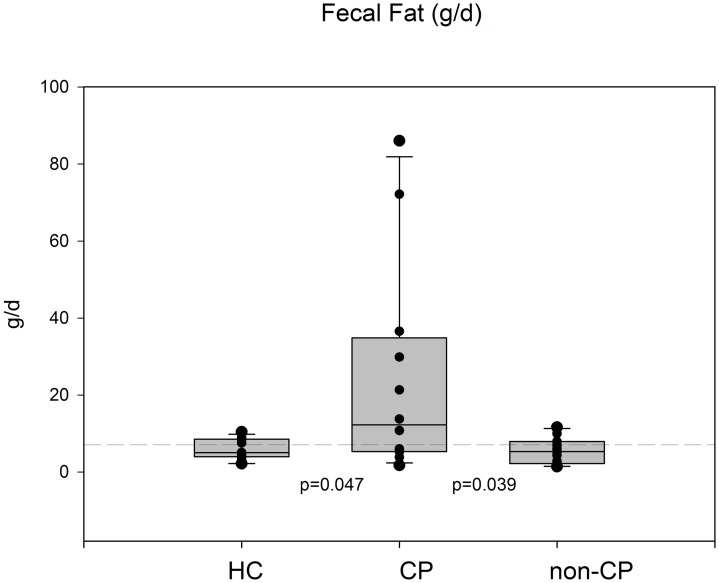
Daily fat output (fecal fat g/d) in median and interquartile range. Cut off after definition in the literature 7 gram per day (dashed line). Significant difference between HC/ CP and non-CP/CP. CP patients have pathologic daily fat output.

**Fig 6:**
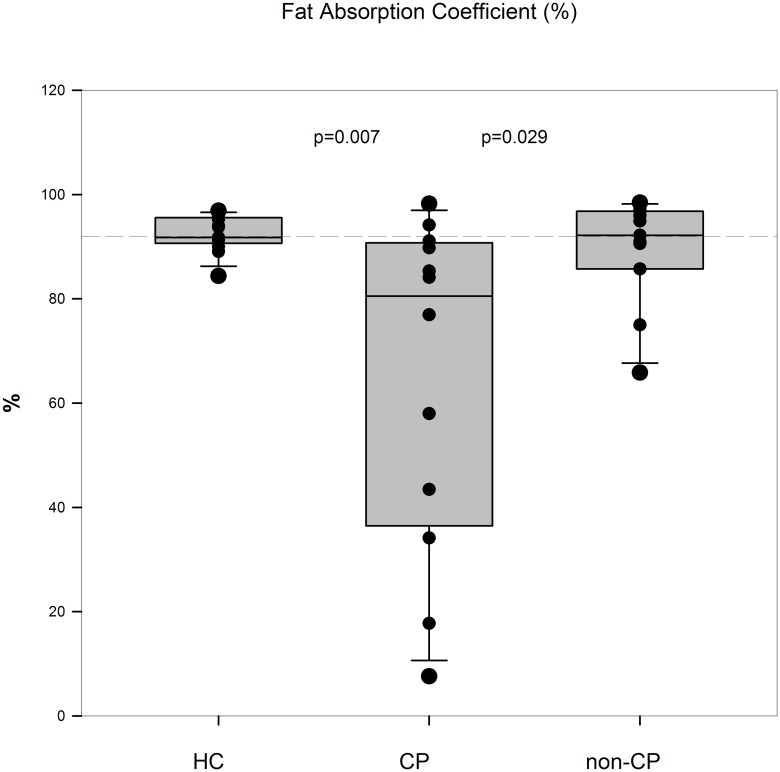
Coefficient of fat absorption. Generally accepted cut off is 90% (dashed line). Reciprocal results as in “A” indicate no additional information of fat absorption. CP patients have significant lower fat absorption than healthy controls and non-CP patients. HC: healthy controls; CP: chronic pancreatitis; non-CP: patients not scoring for chronic pancreatitis.

As expressed in Fig 5, median fecal fat was higher in CP than in non-CP/HC (p = 0.039/p = 0.047). Similarly, in Fig 6 median fat absorption coefficients were lower in CP than in non-CP/HC (p = 0.029/p = 0.007). We did not find any difference between the HC and non-CP group in any of the parameters.

## Discussion

We found that the correlation coefficient between fecal fat and fat absorption coefficient was good regardless of a non-adjusted fat intake in the collection period. Furthermore, we found that the diagnostic performances for fecal fat and CFA were equal when compared to a PEI standard. Except for a diet, containing extreme low or high fat we argue that standardized food ingestion with defined quantity of fat is not necessary to get reliable results on fecal fat. When we stratified the groups according to registered fat intake, we still found that the correlation between the parameters in the group with a fat intake outside the recommended range was comparable to the group with intake within the recommended range of 80 to 120 g/d[8]. Only very low (<40g/d) or very high (>160 g/d) fat intake influenced correlation over the whole range negatively. We conclude that intake of exact 100 g fat per day is not mandatory to get reliable information about quantification of fat malabsorption. These results are contradictory to old dogmas which emphasize the need of standardized fat intake before quantification of fecal fat[23]. As Hart and Conwell discuss, non-standardized fat intake is not a major problem in fecal fat quantification and it is not necessary to admit patients to hospital for performance of the test[5]. Moreover, our results indicate that documentation of nutrients and calculation of coefficient of fat absorption may be dispensable. Our conclusion is supported by Thorsgaard; he described that a fat balanced diet is not necessary in quantification of fecal fat as there are only two studies which state the opposite[24]. We argue that performance of fecal fat analysis without standardized food intake is possible. The focus on patient advice to collect feces correctly without changing eating habits reduces the need for instructions and simplifies the test procedure for the test subjects significantly.

In recent literature, the most cited mean for fat absorption coefficient for healthy controls is 93.5% in a study with 10 healthy subjects[25]. We studied 13 HC and got a mean of 92.4%. Interestingly, six of 13 subjects did not reach intake of 80 g fat per day. Still the mean fat absorption coefficient was comparable to the results achieved in the study by Borowitz[25]. This further supports our hypothesis that strict adjustment of fat intake during the test period is not mandatory.

Interestingly we did not get a correlation factor of one between daily fecal output and coefficient of fat absorption. This phenomenon may be explained by fecal excretion of non-dietary fat. On a diet without lipids fat excretion can be 1–4 g/d[26].

### Limitations

Data were studied retrospectively. Consecutively, a direct comparison of two sample periods on standardized and non-standardized nutritional intake was not feasible. We tried to overcome this limitation by stratifying the groups according to whether fat intake was within or outside the recommended range of 80–120 g/d[8]. The correlations and diagnostic accuracies were good in both groups. Excluding outliers with extreme low (<40g/d) or high values (>160 g/d) in the non-standardized intake group improved the correlation between the parameters in this group further.

Cautious interpretation of the combination of normal fecal fat excretion with low or high fat intake (<40g/d or >160 g/d) has to be taken into consideration. To overcome this pitfall, we recommend asking patients about dietary habits when they receive instruction for stool collection. Within the range from 40g/d to 160g/d, there is no additionally benefit in calculating the fat absorption coefficient.

10 of 12 CP patients had exocrine pancreatic insufficiency. Furthermore, the insufficient subjects where of higher age compared to the sufficient. The prevalence of PEI is not representative for the overall population in Europe and North America pat [27–29]. This is not a population study. The patient selection was done to achieve a continuous range from PS to PEI. Due to the natural time cause of the development towards PEI, the population has an age bias. Reduced intestinal absorptive capacity in the higher age group could be of consequence. However, we argue that implications from PEI dominates as a cause of malabsorption, and the effects of age are of minor importance in the final interpretation of the stool parameters.

The clinical symptoms from steatorrhea are unpredictable. We did not include fecal weight, stool frequency or other clinical characteristics of steatorrhea in this study [7,30,31].

We used a multimodal scoring system for chronic pancreatitis after Layer. Secretin stimulated bicarbonate concentration in duodenal juice defined PEI. Under this condition, the CP group showed less depleted values for daily fecal fat output and coefficient of fat absorption. Fecal fat parameters are less accurate to classify the three study groups. Low sensitivity for fecal fat in the diagnoses of pancreatic exocrine insufficiency is well known[32]. The poor sensitivity can be explained by the following factors. Firstly, from the classical studies of Di Magno we know that steatorrhea is a late sign of PEI[33]. Therefore, comparing parameters of fecal fat -output to a presumable more sensitive standard for PEI like a direct function test, will implicate low sensitivity. The same phenomenon will also withhold when comparing to the CP diagnosis, where the sensitivity of the parameter for early PEI decides the all over sensitivity for the diagnosis in patients with early or mild failure.

In 5 of 13 healthy controls daily fat output was between 8 and 10 g/d. Historically, the cut-off of fecal fat was calculated in the first half of the 20^th^ century, but nutrition has changed considerably since then [34]. To our knowledge, there is no recent study to explore this topic. We suggest that the classical limit of 7g/d is not an absolute threshold. Interpretation of fecal fat testing is only acceptable in the context of clinical information. Other authors argue that diarrhea in patients not suffering from malabsorption can augment fat output substantially and suggest three categories of fecal fat output: Up to 7g/d normal, 7–14 g/d malabsorption of various reasons, >14 g/d pancreatic malabsorption[4,20]. Additional testing of pancreatic function to differentiate pancreatic malabsorption from non-pancreatic malabsorption can address this issue.

A number of 36 study subjects may not be great enough to generalize our findings. An analysis of subgroups is not possible. However other studies have similar or less study participants as in our project and remain as cornerstones in today’s discussions about the topic of our project [21,35]. A prospective crossover study with a larger number of different patient groups and healthy controls may solve this problem.

### Clinical implications

Our simplified procedure of fecal fat determination allows the patients to retain the eating habits, meaning that patients do not have to standardize fat intake during the stool collection period. We believe that this will considerably increase the patient’s compliance towards the test.

Secondary, in the evaluation of pancreatic exocrine function, we tend to compare the tests looking for the best performing PEI parameter. In this setting, the old fecal fat malabsorption tests have lost terrain due to inferior accuracy in the phase of early exocrine failure. In this study, we calculated different PEI parameters. In a detailed evaluation of exocrine failure, the assessment of different aspects of pancreatic exocrine function may be helpful. By combining several tests described in this study, we are able to evaluate the different axes of PEI: duodenal bicarbonate from EST evaluates the ductal axis, fecal elastase evaluates the acinar axis and fecal fat parameters reflect the ability to fat absorption.

High fecal fat output in patients with normal direct and indirect pancreas function tests most probably reflect enteral and not pancreatic fat malabsorption. In this setting, malabsorption parameters like fecal fat measurement are useful tools. Simplifications of these tests may increase availability in clinical use.

## Conclusion

We found good correlation between fat absorption coefficient and fecal fat. Adding knowledge of nutritional intake by using the fat absorption coefficient gave no further improvement in clinical diagnosis of fat malabsorption. We argue that it is not necessary to change eating habits before stool collection and fecal fat quantification.

Finally, we advocate that the additional information on fat malabsorption in PEI may justify keeping fecal fat parameters as a part of the available test repertoire in combination with other available tests for exocrine pancreatic function. The combination of different test axes for exocrine pancreatic function deepens the understanding of different aspects of PEI in pancreas diagnostics.

## Supporting Information

S1 Table Patient groups, original dataset. (XLSX)

## Abbreviations

CP: chronic pancreatitis
HC: Healthy controls
PEI: pancreatic exocrine insufficiency
